# Digital Exclusion Among People Experiencing Homelessness and Residents of Urban Communities in Brazil: Cross-Sectional Study

**DOI:** 10.2196/77124

**Published:** 2026-01-12

**Authors:** Ariela Fehr Tártaro, Dulce Gomes, Thaís Zamboni Berra, Reginaldo Bazon Vaz Tavares, Yan Mathias Alves, Letícia Perticarrara Ferezin, Antônio Carlos Vieira Ramos, Nathalia Zini, Maria Eduarda Pagano Pelodan, Marcela Antunes Paschoal Popolin, Ricardo Alexandre Arcêncio

**Affiliations:** 1 Escola de Enfermagem de Ribeirão Preto Universidade de São Paulo Ribeirão Preto Brazil; 2 Departamento de Matemática Escola de Ciências e Tecnologia Universidade de Évora Évora Portugal; 3 Unidade Acadêmica de Passos Universidade do Estado de Minas Gerais Passos Brazil

**Keywords:** digital health, intersectionality, COVID-19, information-seeking behavior, internet

## Abstract

**Background:**

The COVID-19 pandemic amplified digital divides in Brazil, restricting vulnerable groups’ online access to health information and preventive guidance, with limited intersectional analyses of these inequities.

**Objective:**

This study aimed to investigate inequalities in digital exclusion and access to online COVID-19 information among people experiencing homelessness and residents of urban communities in Brazil by using an intersectional multilevel analysis.

**Methods:**

A cross-sectional study (2021-2023) involving 2652 participants (n=1353, 51% experiencing homelessness and n=1299, 49% from urban communities across 26 state capitals) was conducted using the adapted COVID-19 Social Thermometer questionnaire administered via face-to-face interviews. Multilevel analysis of individual heterogeneity and discriminatory accuracy examined 115 intersectional strata (gender, race and ethnicity, schooling, income, and Brazilian Unified Health System use) with online COVID-19 information seeking as the binary outcome; multilevel logistic models estimated additive effects and between-strata variance.

**Results:**

Most participants were men (1600/2652, 60.3%), self-identified as Black or Brown individuals (1942/2652, 73.2%), and were Unified Health System users (2433/2652, 91.7%) without private insurance (2469/2652, 93.1%). Over one-third (905/2652, 34.1%) had no formal schooling; 62.4% (1655/2652) reported low income. A total of 39.2% (1040/2652) sought online COVID-19 information. Being a woman (odds ratio [OR] 1.49, 95% CI 1.13-1.97), higher schooling (OR 1.78-5.59, 95% CI 3.52-8.88), and higher income (OR 2.37-4.54, 95% CI 2.59-7.93) showed a stronger association with online COVID-19 information seeking; public health system use was not associated with the outcome (OR 0.92, 95% CI 0.64-1.33). Predicted probabilities ranged between 14% and 85% across 115 strata, with the lowest among Black or Brown men (no schooling or low income) and the highest among women and higher schooling or income. The intersectional analysis (n=2405) null model showed 24% between-strata variance; the full additive model reduced it to 1% (proportional change in variance=97%).

**Conclusions:**

Intersectional analysis reveals structural informational exclusion driven by additive disadvantages in schooling, income, and gender among participants, calling for digital inclusion policies, critical health literacy programs, and equitable multichannel communication strategies to address persistent COVID-19 information seeking disparities.

## Introduction

The COVID-19 pandemic exacerbated structural inequalities in health and information systems, highlighting barriers to accessing reliable content [1,2]. In Brazil, the unequal distribution of digital infrastructure, combined with low health literacy, a phenomenon known as the digital divide, undermined the ability of vulnerable social groups to access and apply relevant information for adopting preventive measures [3,4].

Digital exclusion refers to the inability of individuals or groups to fully participate in digital society due to barriers in access, skills, or use, resulting in limited benefits from online services such as health information [5].

In the COVID-19 context, it manifested as restricted access to online pandemic guidance, exacerbated by 3 layers: physical or material access (device or internet costs), skills (digital literacy), and outcomes (effective information seeking) [5]. During lockdowns, services shifted online, excluding people in vulnerable situations without smartphones (eg, 22% of UK adults lacked basic digital skills before the pandemic), creating “informational exclusion” in which low-income, low-literacy groups missed preventive advice, worsening health inequities [6].

Despite growing evidence of the digital divide in Brazil, most studies focus on the general population or on socioeconomically disadvantaged households and rarely include people experiencing homelessness or residents of urban communities as specific populations of interest [4-9]. Furthermore, previous work has typically analyzed social dimensions such as gender, race, educational level, income, and use of health services separately rather than examining how these characteristics intersect to shape digital exclusion and access to online COVID-19 information.

By applying an intersectional multilevel analysis to people experiencing homelessness and residents of urban communities in Brazil, this study addresses this gap and provides new evidence on how overlapping social positions jointly influence inequalities in digital exclusion.

From a methodological perspective, few studies have used multilevel analysis of individual heterogeneity and discriminatory accuracy (MAIHDA), a statistical approach that estimates the effects of specific social combinations on outcomes and can overcome several limitations of traditional additive models [10,11]. Grounded in Black feminist scholarship, this approach provides robust tools for measuring structural inequality [12-14].

In this context, the COVID-19 pandemic presents a critical opportunity to identify patterns of digital and informational exclusion that, if not addressed, are likely to persist and deepen in future public health emergencies. Therefore, this study aimed to investigate inequalities in digital exclusion and access to online COVID-19 information among people experiencing homelessness and residents of urban communities in Brazil using an intersectional multilevel analysis to examine how overlapping social characteristics jointly shape these disparities.

## Methods

### Study Design

This was a cross-sectional study guided by the STROBE (Strengthening the Reporting of Observational Studies in Epidemiology) guidelines [15,16].

### Study Setting

This study was conducted in all 26 Brazilian state capitals and the Federal District, covering the 5 major regions of the country: north, northeast, central-west, southeast, and south. This geographic coverage was defined to encompass diverse urban and regional contexts considering Brazil’s socioterritorial diversity and the deep structural inequalities that characterize the country [17,18].

Brazil has a total area of approximately 8.5 million km^2^ and an estimated population of 203 million [19]. The target population of this study included people experiencing homelessness and residents of urban communities to capture their experiences and vulnerabilities during the COVID-19 pandemic.

The selection of these groups is justified by historical inequalities in access to basic rights, including health care, housing, and information, which were exacerbated during the pandemic [20,21]. It is estimated that the country has approximately 281,400 individuals experiencing homelessness and approximately 16.4 million residents of urban communities, representing 8.1% of the national population [22,23].

### Study Population and Sample

Inclusion criteria were Brazilian nationality, age of ≥18 years, and either residence in an urban community or living in a situation of homelessness. For the intersectional analysis, participants who did not provide responses to key variables, namely, gender, race and ethnicity, income, place of residence, use of the Unified Health System (SUS; *Sistema Único de Saúde* in Portuguese), and information-seeking behavior related to COVID-19, were excluded. This represented 9.3% (247/2652) of the total sample.

Urban communities refer to densely populated territories characterized by unregulated growth and inadequate infrastructure. In contrast, people experiencing homelessness are those without conventional housing, living in public spaces such as sidewalks, squares, and abandoned buildings and exposed to multiple forms of vulnerability [24].

Sampling was sequential, where participants were invited to take part as they were approached in public spaces, shelters, hostels, boarding houses, and urban communities provided they were willing to take part and gave informed consent [25].

Due to the unique characteristics of each of Brazil’s macroregions, the sample did not have a fixed size for each subgroup. Therefore, we applied the standard formula for simple random sampling with finite populations following classic references for opinion polls, epidemiological research, and surveys [26]. Specifically, the Cochran formula for infinite populations is as follows:



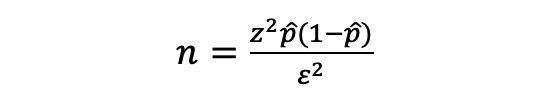



The adjusted Cochran formula for finite populations is as follows:



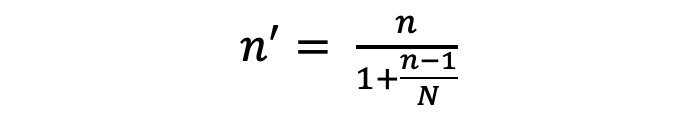



In these formulas, *z* is the percentile of the standard normal distribution, ε is the margin of error, *N* is the population size, and 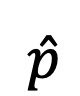 is the estimated population proportion. On the basis of the calculation for finite populations, a minimum sample size of 385 individuals was adopted for the vulnerable population considering a random margin of error of 5%, a confidence level of 95%, a statistical power of 80%, and a variance of 50%. Additionally, a 10% increase was calculated to account for possible sample losses [26,27].

### Data Collection Instrument

The instrument used for data collection was the COVID-19 Social Thermometer: Social Opinion questionnaire, originally developed by the National School of Public Health at NOVA University Lisbon and later adapted and validated for the Brazilian context using the Delphi method. The questionnaire comprises 141 variables, including structured questions formatted as checklists, multiple-choice items, and Likert-scale responses [28].

To ensure data integrity and security, the questionnaire was hosted on REDCap (Research Electronic Data Capture; Vanderbilt University), a secure, web-based platform widely used by academic institutions worldwide for the collection and management of clinical and epidemiological research data. The REDCap version used in this study was installed at the University of São Paulo (USP) Ribeirão Preto campus and includes features that ensure response traceability and internal consistency [29].

### Data Collection

Data collection was conducted between 2021 and 2023 across multiple urban settings characterized by high social vulnerability. Participant mobilization took place through a coordinated network of professionals from research institutions, universities, civil society organizations, and social movements, which facilitated access to different territories and ensured broad geographic coverage.

Participants were recruited as they were encountered in the field following predefined routes and schedules established by the research team in collaboration with local partners. Recruitment took place in public spaces (such as parks, squares, and streets), shelters, social assistance centers, hostels, boarding houses, and urban communities.

Among people experiencing homelessness, interviewers circulated through designated areas previously identified by local teams familiar with the territory and the population. Recruitment followed a standardized protocol: individuals were approached individually, informed about the study, and invited to participate if they met the eligibility criteria and demonstrated willingness to participate. This procedure also ensured participant safety and adherence to ethical standards.

In urban communities, recruitment occurred with the support of local partners in each city, who acted as intermediaries. These groups identified the most appropriate locations and times for conducting interviews, facilitated safe entry into the territory, and guided the research team toward areas with greater resident flow. This collaboration contributed to a systematic recruitment approach and enhanced both the feasibility and safety of fieldwork.

All interviews were conducted face-to-face using mobile devices (tablets or smartphones) by interviewers who were previously trained and exclusively assigned to the project. Training emphasized consistent administration of the questionnaire, standardized participant approach techniques, and the reduction of potential measurement bias. Each interview lasted approximately 20 to 30 minutes and was conducted only once, a strategy necessary due to the high mobility of people experiencing homelessness.

### Study Variables

The outcome variable in this study was the search for information about COVID-19 on the internet, operationalized as a dichotomous variable (“yes” or “no”). In the questionnaire, the question used was as follows: “Which sources of information do you use to stay informed about covid-19?” Among the 8 available response options, the category “internet” was isolated as the main outcome indicator. Participants who selected this option were classified as “yes,” and those who did not select it were classified as “no.”

The internet was selected as the outcome variable due to its central role as a mediator of access to information during public health crises. During the pandemic, the web became the primary channel for disseminating health-related content. However, its use depends on physical access, connectivity, digital literacy, and information seeking skills. Thus, internet use constitutes not only an informational practice but also an indirect marker of structural inequality. By analyzing this variable in isolation, the aim was to identify social profiles with a lower propensity to rely on this resource, particularly among vulnerable populations in territories historically marked by digital exclusion.

To examine differences in information-seeking behavior across dimensions, five socioeconomic characteristics were used to construct intersectional strata: (1) gender (man, woman, or other); (2) race or ethnicity (White; Black or Brown; or other, comprising East Asian and Indigenous identities); (3) household income (low income [no income and less than 1 minimum wage per month], 1 to 2 minimum wages per month, 2 to 3 minimum wages per month, and more than 3 minimum wages per month); (4) schooling (no schooling, basic education, secondary education, and higher education); and (5) use of the SUS (yes and no), the Brazilian public and universal health system, constitutionally guaranteed and responsible for comprehensive health care delivery to the population [30]. Race/ethnicity was self-reported and categorized according to the Brazilian Institute of Geography and Statistics (IBGE) as White, Black, Brown (parda), Asian, and Indigenous.

For race and ethnicity, the categories “other,” “East Asian,” and “Indigenous” were grouped into a single category due to their very low frequency in the sample. This procedure resulted in 3 analytically robust categories: White, Black or Brown, and “other.” A similar limitation occurred for the gender variable, which was collected through self-identification with the response options “woman,” “man,” and “other,” the latter allowing participants to specify another gender identity, including transgender identities. However, no participant selected the “other” option in the final dataset. Consequently, although the study design allowed for the identification of gender diversity, the empirical distribution did not include respondents outside the “woman” or “man” categories.

Furthermore, it is important to highlight that the minimum wage in Brazil varied between R$1100 (US $197.68) and R$1320 (US $237.22) over the course of the study. These variables (sex, race/ethnicity, schooling, household income, and use of the public health system) were selected based on the literature on inequalities in access to information with the goal of analyzing differences in information-seeking behavior across distinct social groups.

### Data Analysis

We used the MAIHDA approach within an intersectional framework [11]. Following the 2-model intersectional MAIHDA approach described by Evans et al [10], the analysis was designed to estimate inequalities across social strata, quantify the role of additive main effects, and detect potential intersectional interaction effects.

An intersectional strata matrix was constructed based on the combination of 5 sociodemographic and health-related dimensions: gender (2 categories), race and ethnicity (3 categories), schooling (4 categories), family income (4 categories), and use of the public health system (2 categories).

This resulted in 192 possible intersectional strata (2 × 3 × 4 × 4 × 2) [10,11,31-33]. Among these, 115 intersectional strata were represented in the study sample and constituted the analytical strata. The selection of these combinations was constrained by data availability but achieved the maximum feasible level of intersectional detail.

Variable selection was guided by the premise that inequalities may exist in access to information relevant to coping with COVID-19. On the basis of the matrix, an intersectional MAIHDA model was conducted, with individual-level data nested within intersectional strata. To estimate the probability of seeking COVID-19–related information, we applied a sequence of 2 multilevel logistic regression models [10,11].

The null model (model 1) included only a random intercept for each intersectional stratum with no fixed covariates. From this model, the between-stratum variance (σᵤ^2^) was estimated, and the variance partition coefficient (VPC) was calculated on the latent scale assuming a fixed residual variance (approximately σₑ^2^=3.29) according to the latent response approach for logistic models. The VPC represents the proportion of the total variance attributable to differences between strata. We also calculated the area under the receiver operating characteristic curve (AUC) to assess the discriminatory accuracy of the strata in predicting internet use [10,11,31-33].

The second model (model 2) included all variables defining the intersectional strata (race and ethnicity, schooling, family income, and use of the public health system) as fixed effects at level 2 while retaining the stratum random intercept. This model allowed us to break down the between-stratum variance into a component explained by additive main effects and a residual component attributable to intersectional interaction effects [10,11,31-33].

The proportional change in variance (PCV) was calculated to quantify the reduction in between-stratum variance relative to the null model. Its complement (1−PCV) represents the portion of residual variance that may be attributed to intersectional interactions. Additionally, predicted probabilities were estimated for each stratum and broken down into an additive component (based on fixed effects) and an interaction component (the difference between the total predicted probability and the additive component). This allowed for the identification of strata with meaningful deviations from additivity [10,11,31-33].

The AUC was interpreted following the work by Axelsson Fisk et al [11]. In MAIHDA models, the AUC is treated as a comparative measure of the strata’s ability to discriminate the outcome, avoiding conventional absolute thresholds for predictive classification. The AUC ranges from 50% (no discriminatory accuracy) to 100% (perfect discriminatory accuracy), reflecting how well intersectional strata distinguish between individuals with and without the outcome [10,11,31-33].

All models were estimated using the maximum likelihood estimation method via the *lme4* package (*lmer* and *glmer* functions) in the R software (version 4.4.0; R Foundation for Statistical Computing) [34].

### Ethical Considerations

This study was approved by the Research Ethics Committee of the Ribeirão Preto School of Nursing at USP, with certificate of submission for ethical appraisal (57933622.4.1001.5393). The entire investigation was conducted in accordance with resolution 466 of December 12, 2012, of the National Health Council considering the relevant ethical and scientific foundations. Before participation, all individuals were informed about the purpose of the study, their rights, and the voluntary nature of participation. Participants received no compensation for participation. The informed consent form was read and explained to participants, and only those who agreed and provided written informed consent were interviewed. For participants who were unable to read or write, consent was obtained verbally and documented through a fingerprint signature. Participant confidentiality was ensured throughout the study. Personal information obtained during data collection was stored securely in encrypted REDCap servers hosted at USP, with access limited to authorized members of the research team. Before any analytical procedures were performed, all datasets were anonymized, and any variables that could identify individuals were removed. Statistical analyses were conducted exclusively with deidentified data, and no results are presented in a manner that could allow for the reidentification of participants.

## Results

### Descriptive Analysis

A total of 2652 individuals participated in the study (Table 1), of whom 1353 (51%) self-identified as people experiencing homelessness and 1299 (49%) were residents of urban communities. Most participants were men (1600/2652, 60.3%), self-identified as Black or Brown individuals (1942/2652, 73.2%), were uninsured (2469/2652, 93.1%), and used the Brazilian SUS (2433/2652, 91.7%). A considerable proportion of participants had no formal education (905/2652, 34.1%) and reported low income (1655/2652, 62.4%). Regarding the use of the internet to seek information about COVID-19, most (1612/2652, 60.8%) stated that they did not use this source.

**Table 1:** Socioeconomic characteristics of vulnerable populations seeking online information about COVID-19 (Brazil, 2021-2023; N=2652).

Variable			Values, n (%)
**Participant category**			
	People experiencing homelessness	1353 (51)	
	People residing in urban communities	1299 (49)	
**Gender**			
	Man	1600 (60.3)	
	Woman	1001 (37.7)	
	No answer	51 (1.9)	
**Race or ethnicity**			
	Black or Brown	1942 (73.2)	
	White	601 (22.7)	
	Other	109 (4.1)	
**Schooling**			
	No schooling	905 (34.1)	
	Basic education	726 (27.4)	
	Secondary education	871 (32.8)	
	Higher education	148 (5.6)	
**Family income**			
	Low income	1655 (62.4)	
	1 to 2 minimum wages	570 (21.5)	
	2 to 3 minimum wages	157 (5.9)	
	More than 3 minimum wages	94 (3.5)	
	Did not know	176 (6.6)	
**Use of the public health system**			
	Yes	2433 (91.7)	
	No	219 (8.3)	
**Health insurance**			
	Yes	172 (6.5)	
	No	2469 (93.1)	
	No answer	11 (0.4)	
**Seeking COVID-19 information online**			
	Yes	1040 (39.2)	
	No	1612 (60.8)	

### MAIHDA Analysis

For the intersectional analysis using the MAIHDA approach, 2405 participants were included after applying eligibility criteria. Table 2 presents the results from the hierarchical logistic models used to estimate the likelihood of seeking information about COVID-19 on the internet based on intersectional strata defined by sociodemographic characteristics.

**Table 2:** Intersectional multilevel analysis of individual heterogeneity and discriminatory accuracy results with parameter estimates from logistic models predicting seeking online information about COVID-19 among vulnerable populations in Brazil (2021-2023).

				Model 1		Model 2
**Fixed effects: regression coefficients, OR^a^ (95% CI)**						
	Intercept		1.02^b^ (0.78-1.31)		0.19^b^ (0.14-0.25)	
	**Gender**					
		Man (reference)	—^c^		—	
		Woman	—		1.49^b^ (1.13-1.97)	
	**Race or ethnicity**					
		Black or Brown	—		—	
		White (reference)	—		1.05 (0.82-1.34)	
		Other	—		1.35 (0.82-2.24)	
	**Schooling**					
		No schooling (reference)	—		—	
		Basic education	—		1.78^b^ (1.32-2.41)	
		Secondary education	—		3.37^b^ (2.53-4.49)	
		Higher education	—		5.59^b^ (3.52-8.88)	
	**Family income**					
		Low income (reference)	—		—	
		1-2 minimum wages	—		2.37^b^ (1.84-3.06)	
		2-3 minimum wages	—		2.85^b^ (1.87-4.34)	
		More than 3 minimum wages	—		4.54^b^ (2.59-7.93)	
	**Use of the public health system**					
		Yes (reference)	—		—	
		No	—		0.92 (0.64-1.33)	
**Summary statistics**						
	Variance partition coefficient (%)		24		1	
	Proportional change in variance (%)		—		97	
	Area under the receiver operating characteristic curve		0.74		0.73	

^a^OR: odds ratio.

^b^Statistically significant at *P*<.05.

^c^Not applicable.

Model 1 (null) showed a VPC of 24%, indicating that nearly a quarter of the total variation in information-seeking behavior lay between social strata; that is, between-group inequality was substantial. The AUC was 0.74.

Model 2, which included the individual components of the strata as additive effects, reduced the VPC to 1%, with a PCV of 97%. This means that almost all the inequality observed between strata can be explained by additive effects of gender, race, educational level, income, and age. The AUC remained practically unchanged (0.73), suggesting that incorporating additive effects did not significantly improve the predictive capacity of the model.

Women were more likely to seek information online than men (odds ratio [OR] 1.49, 95% CI 1.13-1.97), representing a 49% increase in odds relative to the reference group.

Schooling was positively associated with the outcome. Compared to individuals with no formal schooling, those with primary schooling had a 78% higher likelihood of seeking information (OR 1.78, 95% CI 1.32-2.41), those with secondary schooling had a 237% increase (OR 3.37, 95% CI 2.53-4.49), and those with higher education had a 459% increase (OR 5.59, 95% CI 3.52-8.88).

Household income was also positively associated with information seeking. Compared to participants in the lowest income category, those earning between 1 and 2 minimum wages had an OR of 2.37 (95% CI 1.84-3.06), those earning between 2 and 3 minimum wages had an OR of 2.85 (95% CI 1.87-4.34), and those who earned more than 3 minimum wages had an OR of 4.54 (95% CI 2.59-7.93). Use of the public health system was not significantly associated with the outcome (OR 0.92, 95% CI 0.64-1.33).

Comparison between models revealed a substantial reduction in between-strata variance: the VPC dropped from 25% in the null model to 0.01% in the full model, resulting in a PCV of 97%. The VPC of 25% indicates that one-quarter of the variance in information-seeking behavior was attributable to intersectional groupings. This finding suggests that most of the explained variance was due to the additive effects of individual variables rather than intersectional interactions. The AUC for model 2 was 0.73, maintaining a similar discriminatory performance to that of the null model.

**Figure 1:**
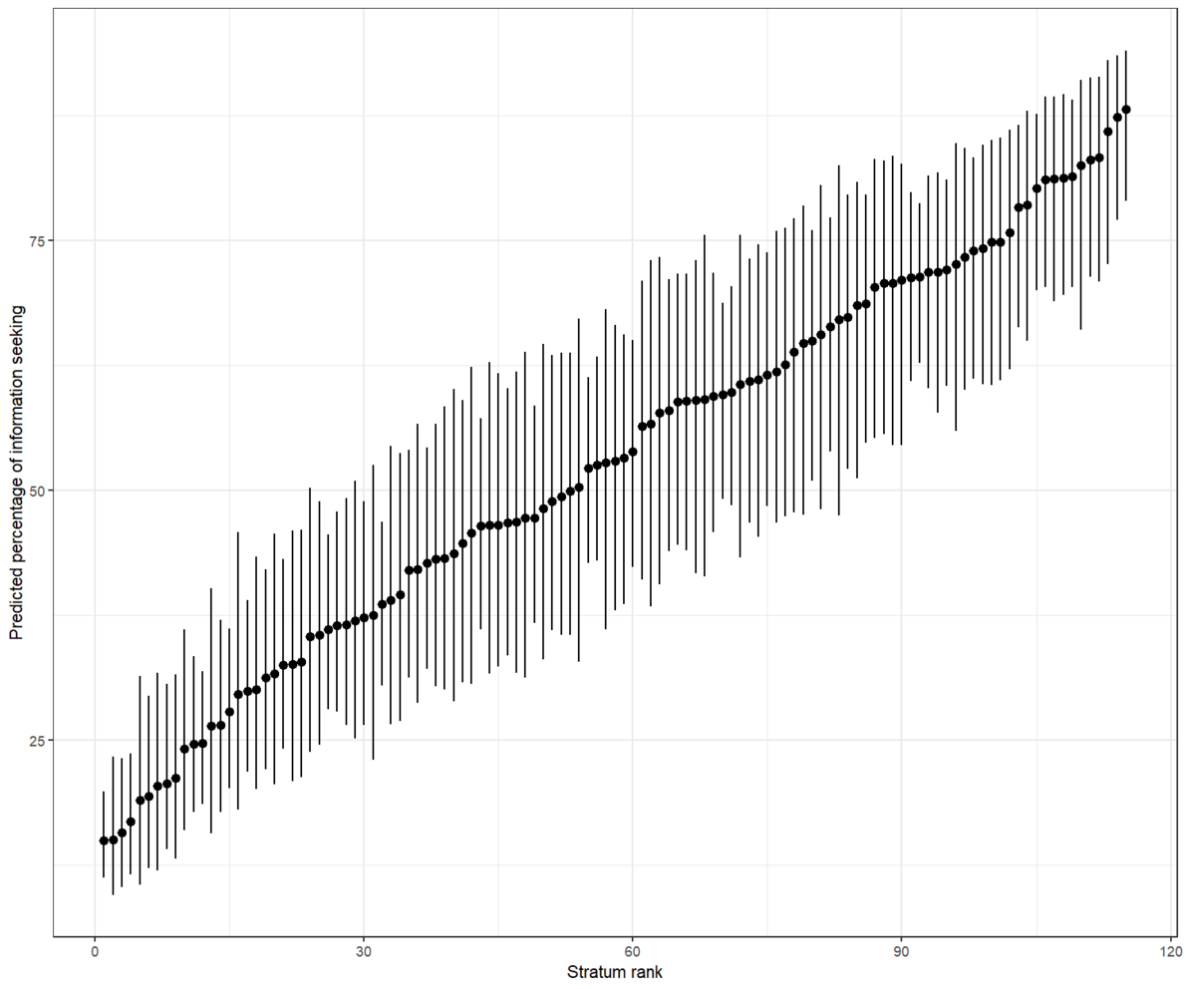
Predicted probabilities of seeking COVID‑19 information across intersectional strata (null model) in Brazil in 2021-2023.

Figure 1 shows the predicted probabilities of seeking COVID-19 information across intersectional strata (null model). Strata are ranked from the lowest to the highest predicted probability. Predicted values range from approximately 15% to nearly 90%, revealing a wide gradient of intersectional inequality.

The continuous distribution—without discrete clusters—suggests cumulative and overlapping disadvantage. Wider CIs for some strata reflect smaller sample sizes and greater estimation uncertainty. Because the null model captures both additive and interactive components, this distribution represents the overall pattern of intersectional heterogeneity in information-seeking behavior.

Table 3 presents the 5 intersectional strata with the lowest and highest predicted probabilities of seeking information about COVID-19 online based on estimates from the null model (model 1). The strata are organized in ascending order of predicted probability.

**Table 3:** Classification of strata by predicted probability levels (highest and lowest) of seeking online information about COVID-19 among vulnerable populations (null model; Brazil, 2021-2023).

Rank		Stratum ID	Gender	Race or ethnicity	Schooling	Family income	Use of the public health system	n (participants per stratum)	Predicted probability of seeking online information about COVID-19 (95% CI)
**Five strata with the lowest information seeking**									
	1	12111	Man	Black or Brown	No schooling	Low income	Yes	335	14.26 (9.20-23.13)
	2	13111	Man	Other	No schooling	Low income	Yes	19	18.31 (10.80-31.78)
	3	23111	Woman	Other	No schooling	Low income	Yes	8	18.40 (10.80-31.78)
	4	21111	Woman	White	No schooling	Low income	Yes	38	19.48 (11.71-23.84)
	5	12112	Man	Black or Brown	No schooling	Low income	No	53	19.61 (9.91-23.86)
**Five strata with the highest information seeking**									
	110	21321	Woman	White	High schooling	1-2 minimum wages	Yes	35	79.17 (69.54-90.65)
	111	22421	Woman	Black or Brown	Higher education	1-2 minimum wages	Yes	11	79.17 (69.54-90.65)
	113	12421	Man	Black or Brown	Higher education	1-2 minimum wages	Yes	12	80.32 (73.28-93.25)
	114	11441	Man	White	Higher education	More than 3 minimum wages	Yes	8	81.80 (76.58-92.86)
	115	11331	Man	White	Higher education	2-3 minimum wages	Yes	11	84.82 (73.28-93.25)

Among the 5 strata with the lowest predicted probabilities, there was a predominance of men; Black, Brown, or other race or ethnicity; no formal education; low-income households; and use of the public health system. The lowest predicted probability was 14.26% (95% CI 9.2%-23.13%).

In contrast, the 5 strata with the highest predicted probabilities showed an opposite profile: most were women or men with higher education, middle to high income (1-3 minimum wages), and users of the public health system. The predicted probabilities of information seeking in these strata ranged from 79.17% (95% CI 69.54%-90.65%) to 84.82% (95% CI 73.28%-93.25%).

These findings highlight the coexistence of significant intersectional disparities in health information seeking during the pandemic associated with structural markers of vulnerability such as race or ethnicity, educational attainment, income, and access to public health services

## Discussion

### Principal Findings

This study aimed to investigate inequalities in digital exclusion and access to online COVID-19 information among people experiencing homelessness and urban community residents in Brazil using intersectional multilevel analysis. Key findings show that only 39.2% (1040/2652) sought online COVID-19 information, with being a woman, higher schooling, and income strongly linked to higher access across 115 intersectional strata.

The findings also revealed predicted probabilities ranging from 14% (Black or Brown men, no schooling, and low income) to 85% (higher educated, higher income groups), with 97% of the between-strata variance (VPC=24% to 1%) explained by additive effects rather than interactions. The findings confirm structural digital exclusion driven by overlapping gender, schooling, income, race or ethnicity, and SUS use disadvantages, fulfilling the study’s goal of mapping how social positions jointly shaped informational inequities during the pandemic [10,11,32].

The association between higher levels of education and greater use of the internet as a source of health information reflects not only technical access but also, more importantly, the possession of cognitive and informational competencies that support autonomy in health care [35]. Income, in turn, functions as a proxy for time availability, connectivity, access to digital devices, and overall stability, factors that are all essential for sustained online engagement. This underscores the structural nature of digital exclusion [36,37].

The absence of an association between the use of the SUS and online health information seeking suggests that the public health care system still lacks effective digital strategies for informational mediation targeting vulnerable populations. This finding offers a critical lesson for future public health emergencies: institutional digital channels alone are not sufficient. Health communication requires multichannel, territory-based approaches supported by community-based mediation [38].

Notably, all 5 of the strata with the lowest predicted probability of seeking COVID-19 information online were users of the SUS, whereas among the strata with the highest predicted probability, SUS users also predominated. This suggests that the inequality does not lie between those who do or do not use the public health system but rather in how intersectional social characteristics moderate digital engagement even within the same health care system.

By identifying that men with low levels of education and income exhibit the lowest rates of health information seeking online, this study highlights a social group that remains systematically overlooked in communication strategies. This population profile embodies multiple layers of exclusion (informational, economic, and institutional), placing them at constant risk in the face of misinformation and informational disengagement [20,21].

The relationship between gender and health information seeking observed in this study contrasts with international findings that identified greater male engagement in COVID-19–related communicative uses of the internet. While the international study assessed online communication practices such as content sharing and interaction on social networks, our focus was on the active search for health information.

This distinction suggests that gender differences in the digital sphere are not homogeneous but vary according to the type of activity analyzed. These findings also point to an expanded understanding of digital exclusion, which is not limited to physical access but involves symbolic and functional disconnections from the networks through which knowledge circulates. The greater tendency among women to seek health information may be associated with their central role in care networks, indicating the potential for future strategies anchored in female and community leadership [39-41].

This pattern is not exclusive to the Brazilian context. Both national and international studies have shown that groups with lower educational levels and those residing in marginalized or racialized territories consistently exhibit reduced access to and critical use of digital health information [6,7,37,42,43]. In the United States, Suh et al [37] found that communities facing greater socioeconomic vulnerability showed limited increases in online information seeking even during a global crisis. This suggests that exclusion patterns are not only resistant to exceptional circumstances but may, in fact, be exacerbated by them [37].

Moreover, research has shown that overcoming digital exclusion requires more than infrastructure expansion. Public policies must address informational competencies and invest in effective symbolic mediation channels, especially in vulnerable contexts [31,41]. In Brazil, qualitative studies indicate that community leaders and public health agents remain the primary bridges between the population and health information, suggesting that trust and accessibility are as crucial as connectivity [44].

These results engage with international debates on digital health inequities, which highlight how structurally marginalized groups face not only limited physical access but also a drastically reduced capacity to translate information into health action [4]. Such processes intensify during public health emergencies, when the infodemic rather than democratizing access often amplifies existing asymmetries by demanding complex digital literacies and continuous connectivity, 2 factors profoundly shaped by class, gender, and race [37].

Corroborating this picture, studies show that older adults, people with lower educational attainment, and those with low traditional literacy are precisely those who were least able to obtain positive outcomes from internet use during the COVID-19 pandemic [4].

Such identification is strategically valuable for the formulation of proactive public policies that can mitigate disparities before they become irreversible in future crises. Thus, the trends observed during the pandemic should be interpreted as early warnings of chronic vulnerabilities. The adoption of exclusively digital solutions without territorial grounding or community mediation tends to reproduce and even amplify the very asymmetries this study reveals.

Therefore, promoting informational equity is not a peripheral goal but a central tenet of health justice. It is essential to ensure that all social groups not only have access to information but are also able to understand, contextualize, and use it to make informed care decisions. Tackling the infodemic demands integrated approaches that combine digital inclusion, critical literacy, trust networks, and communication strategies sensitive to territorial diversity [2,4].

These findings underscore the urgency of territorialized strategies to combat infodemics, as evidenced by studies in Brazilian favelas in which digital divides during COVID-19 amplified misinformation uptake among low-income residents lacking community mediation [45-47]. Literature on health communication highlights how ungrounded digital interventions exacerbate asymmetries in rural and indigenous groups, reducing trust and engagement due to cultural mismatches [45-47]. Taken together, these findings suggest that future research and interventions should further explore how territorially grounded and socially mediated communication strategies can mitigate informational inequalities during public health emergencies.

This study has limitations. Its cross-sectional design does not allow for causal inferences, and using a single question to measure the outcome may not fully capture the complexity of information-seeking behaviors. In addition, self-reported data are vulnerable to recall and social desirability bias, although the use of a validated instrument, face-to-face data collection, and trained interviewers strengthens the reliability of the findings [28].

There were also important constraints related to measuring gender and race and ethnicity. Although the questionnaire allowed for self-identification beyond binary gender and included detailed racial and ethnic categories, no participant identified as gender diverse or transgender, and very few identified as Indigenous, East Asian, or “other.” This underrepresentation is consistent with evidence that gender and racial and ethnic minority groups are often less visible in surveys involving socially vulnerable populations because of stigma, safety concerns, and structural barriers to participation [48].

From a methodological perspective, the absence of participants outside the “woman” and “man” categories prevented exploration of gender diversity within the intersectional MAIHDA framework. Very small cell sizes in some strata also required grouping categories to preserve statistical stability and confidentiality, which reduced the granularity of intersectional comparisons [10].

Finally, the analysis was limited to participants with complete data for all intersectional variables. While this complete-case strategy was necessary to construct comparable strata, it may have disproportionately excluded individuals in more vulnerable situations, introducing potential selection bias.

We highlight the need for future studies using mixed methods approaches to investigate the meanings attributed to information, the relationship between formal and informal sources, and the impact of community mediation on the development of informational attitudes. Research that integrates qualitative and quantitative methods may offer new insights into the information behavior of vulnerable populations and help guide more inclusive, effective, and sustainable health communication policies.

### Conclusions

Beyond mapping intersectional patterns, this analysis reveals how overlapping social positions (gender, race and ethnicity, schooling, and income) drive structural disparities in online COVID-19 information seeking among people experiencing homelessness and urban community residents in Brazil. These patterns underscore additive disadvantages as the primary mechanism of digital exclusion rather than synergistic interactions, highlighting the cumulative impact of vulnerability markers even within the public health system. Identifying these extreme social profiles enables policymakers to prioritize territory-based digital inclusion strategies, advancing informational equity and health justice in future public health crises. These intersectional disparities reveal that, even within Brazil’s constitutionally guaranteed SUS, a fundamental right under Article 196 of the 1988 Federal Constitution, no vulnerable group should be left behind in accessing essential health information.

## Abbreviations

AUC: area under the receiver operating characteristic curve
MAIHDA: multilevel analysis of individual heterogeneity and discriminatory accuracy
OR: odds ratio
PCV: proportional change in variance
REDCap: Research Electronic Data Capture
STROBE: Strengthening the Reporting of Observational Studies in Epidemiology
SUS: Unified Health System (Sistema Único de Saúde in Portuguese)
USP: University of São Paulo
VPC: variance partition coefficient

## Data Availability

The datasets generated or analyzed during this study are available from the corresponding author on reasonable request.
